# Comparative transcriptomic and proteomic analyses reveal upregulated expression of virulence and iron transport factors of *Aeromonas hydrophila* under iron limitation

**DOI:** 10.1186/s12866-018-1178-8

**Published:** 2018-06-04

**Authors:** Tao Teng, Bingwen Xi, Kai Chen, Liangkun Pan, Jun Xie, Pao Xu

**Affiliations:** 1 Wuxi Fisheries College, Nanjing Agricultural University, Wuxi, 214081 China; 2 Key Laboratory of Freshwater Fisheries and Germplasm Resources Utilization, Ministry of Agriculture, Freshwater Fisheries Research Center, Chinese Academy of Fishery Sciences, Wuxi, 214081 China

**Keywords:** Transcriptomic, Proteomic, Virulence, Iron, *Aeromonas hydrophila*

## Abstract

**Background:**

Iron plays important roles in the growth, reproduction and pathogenicity of *Aeromonas hydrophila*. In this study, we detected and compared the mRNA and protein expression profiles of *A. hydrophila* under normal and iron restricted medium with 200 μM 2,2-Dipyridyl using RNA Sequencing (RNA-seq) and isobaric tags for relative and absolute quantification (iTRAQ) analyses.

**Results:**

There were 1204 genes (601 up- and 603 down-regulated) and 236 proteins (90 up- and 146 down-regulated) shown to be differentially expressed, and 167 genes and proteins that showed consistent expression. Gene Ontology (GO) and Kyoto Encyclopedia of Genes and Genomes (KEGG) enrichment analyses revealed that the differentially expressed genes and proteins were mainly involved in iron ion transport, protein activity, energy metabolism and virulence processes. Further validation of the RNA-seq and iTRAQ results by quantitative real-time PCR (qPCR) revealed that 18 of the 20 selected genes were consistently expressed. The iron-ion absorption and concentration of *A. hydrophila* under iron-limited conditions were enhanced, and most virulence factors (protease activity, hemolytic activity, lipase activity, and swimming ability) were also increased. Artificial *A. hydrophila* infection caused higher mortality in cyprinid *Megalobrama amblycephala* under iron-limited conditions.

**Conclusion:**

Understanding the responses of pathogenic *Aeromonas hydrophila* within the hostile environment of the fish host, devoid of free iron, is important to reveal bacterial infection and pathogenesis. This study further confirmed the previous finding that iron-limitation efficiently enhanced the virulence of *A. hydrophila* using multi-omics analyses. We identified differentially expressed genes and proteins, related to enterobactin synthesis and virulence establishment, that play important roles in addressing iron scarcity.

**Electronic supplementary material:**

The online version of this article (10.1186/s12866-018-1178-8) contains supplementary material, which is available to authorized users.

## Background

*Aeromonas hydrophila* is an opportunistic pathogenic bacterium that is ubiquitous in aquatic environments and causes serious infections worldwide in cultured fishes, amphibians, reptiles, and even mammals [1–4]. The pathogenesis of *A. hydrophila* is multifactorial, causing disease with virulence factors, such as adhesins, cytotoxins, hemolysins, and proteases, and it has the capacity to form biofilms and alter metabolic pathways and gene expression under various host environments [5, 6]. Its virulence expression is closely related to the environment in which the bacteria live (in vivo and in vitro), nutrients, and so on [7]. For example, the nutrient iron deficiency in the host environment has been thoroughly documented as having a pronounced effect on the virulence of pathogens [8].

Iron is an indispensable element of most living cells that is involved in many cellular functions, including electron transportation and oxygen transportation. The quantity of iron has a great impact on biological processes, for instance, iron overload will result in iron toxicity to cellular components [9], especially for DNA damage, owing to the reactions between hydroxyl radicals and other biomolecules [10, 11]. However, iron deficiency can also cause malnutrition cell death in severe cases [12]. In vivo, iron is usually oxidized to an insoluble form due to its special physico-chemical properties, bonding with heme, ferritin, hemoglobin, and transferrin within the cells, and thus is not readily accessible to bacteria [13]. In response to this iron deficiency predicament, microorganisms have evolved a series of sophisticated mechanisms to compete against the host, such as the secretion of siderophores [14], to grab iron from transferrin, hemoglobin, and ferritin and maintain iron dynamic balance for bacterial growth, proliferation, and toxin secretion [15–17]. During the past decades, the bacterial iron acquisition system and virulence have attracted much attention. For example, CaFTR1-mediated iron-uptake was proven to be an important virulence factor of *Candida albicans* [18], iron-responsive transcriptional repressor PerR was required for full virulence in *Staphylococcus aureus* [19], and FeoB was determined to play an important role in Fe acquisition expression of virulence of *Helicobacter pylori* [20].

Pathogenic bacteria virulence factors under iron-restricted growth conditions have previously been published [21–24]. Proteomes and transcriptomes reflect gene expressions from two different levels, and their joint analysis provides more complete expression information about bacteria. Therefore, in this study, an iron stress model was established to maximize the simulation of iron deficiency environment in vivo, and the effects of iron-restricted stress on the growth and virulence of *A. hydrophila* were evaluated comprehensively by combining transcriptome and proteomics data.

## Methods

### Selection of iron chelator concentration and growth of *A. hydrophila*

*A. hydrophila* (NJ-35) was isolated from dead cultured cyprinid in Jiangsu Province, China [25], and kindly provided by Professor Yongjie Liu from the College of Veterinary Medicine, Nanjing Agricultural University, P.R. China. We selected 2,2’-Bipyridyl (Bip) (Sinopharm Chemical Reagent Co., Ltd., Shanghai, China) as the ferrous iron chelating agent because of its high cell membrane permeation and intracellular iron sequestering ability [26–28]. The accuracy and virulence of *A. hydrophila* NJ-35 were confirmed by 16S rRNA gene sequencing (Biological Engineering Technology Co., Shanghai, China) and lab infection assays, respectively. Six concentrations (0, 100, 200, 300, 400, and 500 μM Bip in normal tryptic soy broth medium (TSB; BD; final pH = 7.3)) were set to detect the optimal concentration according to the growth curve of *A. hydrophila* NJ-35. *A. hydrophila* NJ-35 was inoculated in 5 ml of normal TSB and incubated (28 °C, 24 h); bacteria cells were collected via centrifugation, washed three times with PBS, and then diluted to an optical density at 600 nm (OD 600) of 0.01 in 100 mL of normal TSB to culture (180 rpm, 28 °C).

### Sample collection

*A. hydrophila* NJ-35 cells (OD 600 ≅ 0.8) in normal and iron-limited groups were collected by centrifugation (5000 rpm, 4 °C, 10 mins). The pellet was rinsed twice with saline and stored immediately at − 80 °C until further transcriptomic and proteomic analyses. The supernatant was retained, filtered (MILLEX®GP filter unit, 0.22 μm), and frozen at − 20 °C, and it was used for the following proteolytic and hemolytic activity analyses.

### Determination of iron concentration

The atomic absorption spectrophotometry (GB/T 5009.90–2003) method [29] was used to the measure variations in the intracellular iron of *A. hydrophila* NJ-35 in normal and iron-limited groups, as well as the iron concentration in the broth. Samples were analyzed by the Jiangsu Provincial Food Safety Testing Co., Ltd.

### Quantitative transcriptomics (RNA-seq)

#### (i) *RNA isolation and mRNA purification*

Total RNA was purified using an RNAqueous kit (Thermo Fisher Scientific, San Jose, CA, USA) according to the manufacturer’s instructions. The RNA concentration and integrity (RIN) were measured following the previous description of Wang et al. [30]. The mRNA was enriched using a MICROBExpress Kit (Ambion, USA) [31], and determined on Agilent 2100 Bioanalyzer.

#### (ii) *cDNA Synthesis, Illumina sequencing and library construction*

Bacterial mRNA was fragmented using an RNA fragmentation kit (Illumina, San Diego, CA, USA). Double-stranded cDNA was synthesized using SuperScript II Reverse Transcriptase (Invitrogen, Carlsbad, CA) according to the manufacturer’s recommendations. Libraries were prepared with the standard protocol of the TruSeq RNA Sample Prep v2 Low Throughput (LT) kit. Paired-end sequencing was processed by the Hiseq™2000 (Illumina, San Diego, CA, USA) sequencer.

#### (iii) *Bioinformatics Analyses*

The assembled reads were mapped to the complete genome of the *A. hydrophila* NJ-35 strain (http://www.ncbi.nlm.nih.gov/nuccore/CP006870.1). The QC of alignment was produced based on the standard generated by Qin et al. [31]. The gene expression level was calculated using the RPKM method (fragments per kb per million reads) [32]. Differentially expressed genes (DEGs) were identified with EdgeR software [33], and used to generate statistical information such as expression level, fold change, *p*-value and FDR (false discovery rate). The specific filter conditions of DEGs were: log_2_(fold change) ≥ 2, *p* < 0.05 and bcv (biological coefficient of variation) = 0.01.

GO enrichment analyses of DEGs were performed on website (http://www.geneontology.org/). The calculation method, p-value formula and enrichment score were analyzed according to the method reported by Yan et al. [34].

Additionally, the DEGs were subjected to KEGG enrichment analyses [35] to identify their main metabolic pathways. The formula used for calculation was the same as that in the GO analyses.

### Quantitative proteomics (iTRAQ)

#### (i) Protein extraction, quantization, and SDS-PAGE electrophoresis

The extract of whole cellular protein was conducted according to Isaacson et al. [36] with some modification. The bacterial cells pellets were suspended in cooled acetone (1 h, − 20 °C), centrifuged (15,000×g, 15 mins, 4 °C), and dried with a vacuum freeze dryer. The samples were resuspended in cold saturated-phenol (pH 7.5) and shaken (30 mins, 4 °C). The upper phenolic phase was collected by centrifugation (5000×g, 30 mins, 4 °C), 5 volumes of cold 0.1 M ammonium acetate in methanol was added, and then it was stored (1 h, − 20 °C). After centrifugation (5000×g, 30 mins, 4 °C), the pellets were washed and mixed with 2 volumes of ice-cold methanol. The pellets were centrifuged, dried and dissolved in lysis solution (1 h, 30 °C). The supernatants were isolated by centrifugation (15,000×g, 15 mins). The protein concentrations were measured with the BCA method [37], after which they were stored at − 80 °C for iTRAQ analyses. Additionally, 10 μg samples were subjected to 12% SDS-PAGE, visualized and then scanned according to Candiano’s protocol [38].

#### (ii) protein samples preparation and labeling

The filter-aided sample preparation (FASP) method [39] was adopted for enzymatic hydrolysis of the proteins (100 μg). After 50 μL trypsin (50 ng/μL) digestion, peptides were labeled according to the manufacture’s protocol for 8-plex iTRAQ reagent (AB SCIEX, USA).

#### (iii) 2D-LC-MSMS analyses

### RPLC analyses

The dried samples were resuspended with 100 μL buffer A, after which reversed-phase liquid chromatography (RPLC) was employed on an Agilent 1200 HPLC System (Agilent). Separation was conducted according to the method of You et al. [40]. The first segment was collected from 0 to 5 mins, after which each additional segment was collected at a 4.5 min interval for 6–45 min, while the last segment was collected from 46 to 50 mins for a total of 10 segments. Each segment was dried and used for subsequent RPLC-MSMS analyses.

### RPLC-MSMS analyses

In brief, samples were resuspended with Nano-RPLC buffer, filtered through a C18 nanoLC trap column, and a Chromxp C18 column (75 μm × 15 cm, C18, 3 μm 120 Å). The Eksigent nanoLC-Ultra™ 2D System (AB SCIEX) was used to perform the online Nano-RPLC. Triple TOF 5600 system (AB SCIEX, USA) was used to analyze MS data combined with Nanospray III source (AB SCIEX, USA).

#### (iiii) protein identification and quantification

Data were processed with the Protein Pilot Software v. 5.0 (AB SCIEX, USA) against the NCBI database using the Paragon algorithm [41]. The results of protein quantification were obtained by the matching of tandem mass spectrometry (MS) data and theoretical data, and was performed with the search option: emphasis on biological modifications.

An Orbitrap Elite high-resolution mass spectrometer (Thermo Fisher Scientific, USA) was used for ITRAQ quantitative proteomic analyses. Normalized high-energy collision dissociation (HCD) was performed, with the collision energy set at 30%. A protein database search and quantification were performed using Maxquant 1.5.1.0 (Thermo Fisher Scientific, USA). The protein database contained 4119 proteins (https://www.ncbi.nlm.nih.gov/genome/?term=Aeromonas+hydrophila, GCF_000014805.1_ASM1480v1_protein.faa). Oxidation (M) and acetyl (protein N-term) were used as the variable modifications and carbamidomethyl (C) was the fixed modification. The MS/MS tol. (FTMS) was 20 ppm. The protein quantitation, peptides matching and the functional annotations of DEPs were performed according to the method reported by Yao et al. [24].

### Primer design, quantitative real-time PCR (qRT-PCR) validation

All of the sequence-specific primers of the target genes for qRT-PCR analyses were designed using Primer 5.0 based on the obtained fragment (Table 3). The mRNA level of *rpoB* was used as an internal reference because of its stable expression according to Zhang et al. [42].

Total RNA from *A. hydrophila* was extracted using RNAiso Plus (TaKaRa, Japan), and measured using a Nanodrop 2000 (Thermo Fisher Scientific, USA), the RNA concentration of each sample were diluted to 40 ng/μL, and then 2 μg of the total RNA was subjected to the following quantitative analysis with a One Step SYBR® PrimeScript® Plus RT-PCR Kit (TaKaRa, Dalian). Triplicate quantitative assays were performed on each type of cDNA using the ABI 7500 Real-time PCR System (Applied Biosystems, Foster City, CA, USA) and analyzed with the two-standard curve method.

### Proteolytic activity

Proteolytic activity was measured by an azocasein assay method of Swift et al. [43] and Chu et al. [44], with some modifications. Briefly, 150 μL of normal group and iron-limitation group NJ-35 culture supernatants were added to 1 ml of 0.3% azocasein (Sigma, St. Louis, USA) in 0.05 M Tris-HC1 and 0.5 mM CaCl_2_ (pH 7.5), then they were incubated (37 °C, 30 mins) respectively. Precooling trichloroacetic acid (l0%, 0.5 ml) was then added to stop the reaction, after which the samples were allowed to stand for 15 mins at room temperature, then they were centrifuged (12,000 rpm, 10 mins, 4 °C) to remove the precipitate. Next, 500 μL of the supernatants were added to an equal volume of NaOH (1 mol/L). The supernatants (200 μL) were subsequently transferred to a 96-well tissue culture plate, after which the absorbance (OD400) of the supernatant was measured. The proteolytic activity was calculated using the following equation: proteolytic activity = OD_400nm_ sample – OD_400nm_ blank control (normal TSB/iron limitation TSB).

### Hemolytic activity

Hemolytic activity was determined as previously described [45, 46], and sheep blood (Ping Rui Biotechnology, China) was prepared by washing thrice with PBS. Washed sheep blood (10 μL) was added to 490 μL of the experiment supernatants (sample), normal TSB/iron limitation TSB (blank control), 1% (*v*/v) Trinton X-100 (positive control), or PBS (phosphate buffer solution, negative control). After 30 mins of incubation at 37 °C, all of the samples were centrifuged (5000 rpm, 10 mins) at room temperature. The supernatants (200 μL) were then transferred to a 96-well tissue culture plate, after which the absorbance of hemoglobin released for each solution at 540 nm was measured. The percentage of hemolysis was calculated using the following equation: hemolysis (%) = (OD_540nm_ sample - OD_540nm_ blank control)/ (OD_540nm_ positive control Trinton X-100 - OD_540nm_ negative control PBS).

### Lipase activity

Bacterial cells were centrifuged and washed with PBS, after which 5 μL of bacterial fluid was used to inoculate the LB medium containing a 1% mass fraction of Tween 80. Samples were then incubated at 28 °C for 24 h, after which they were observed for lipase production, which was indicated by a white precipitate zone around the colony.

### Motility

The target bacteria were centrifuged and washed with sterilized PBS. Next, 5 μL of bacterial fluid was dropped onto LB semisolid agar plates containing 0.3% agar (to determine swimming ability) and 0.5% agar (to determine swarming motility). The LB plates were subsequently sealed with parafilm and incubated at 28 °C for 24 h (three parallel groups were set up for each group). At the end of the culture period, the migration distance from the colony edge to the colony center was determined. The experiment was repeated three times.

### Infection assays in vivo

A health check was conducted and healthy *M. amblycephala* (50 ± 5 g) were obtained from the Nanquan Experimental Station of the Freshwater Fisheries Research Center (Chinese Academy of Fishery Sciences, China) and acclimatized in circulating water system with thermo-control for 2 weeks before use. Fish were given commercial feed. The water temperature fluctuated between 27.5–28.5 °C, with a pH between 7.2–7.8, and the DO was about 5.5 mg/L.

Strain NJ-35 was inoculated aseptically into normal TSB medium and iron-limitation medium and then incubated for 18 h at 28 °C while shaking at 180 rpm. The artificial challenge experiment was performed as the previous report [47]. To determine the 50% lethal dose (LD_50_) [48], five groups of 20 *M. amblycephala* each were injected intraperitoneally with 150 μL of serial ten-fold diluted bacterial suspensions (1 × 10^9^, 10^8^, 10^7^, 10^6^, and 10^5^ CFU·mL-1 measured by turbidimeter (Yue Fung Instrument Co., Ltd., Shanghai, China)), which were diluted with 0.9% saline. Next, an experimental group and a control group were injected intraperitoneally with 150 μL *A. hydrophila* (LD_50_) iron-limited and *A. hydrophila* (LD_50_) basal, respectively, and the virulence was compared. Three replicate tanks per challenge isolate (containing 20 fish each) were used to calculate survival (from a total of 60 fish per isolate). The mortality of the fish of experimental groups and control groups were monitored (7 days), and the activity and behavior were recorded daily; pathogenic bacteria were isolated and identified from the lesion tissues of dead fish as the judging standard.

## Results

### Growth of *A. hydrophila* under different iron-limitation medium

**Fig. 1:**
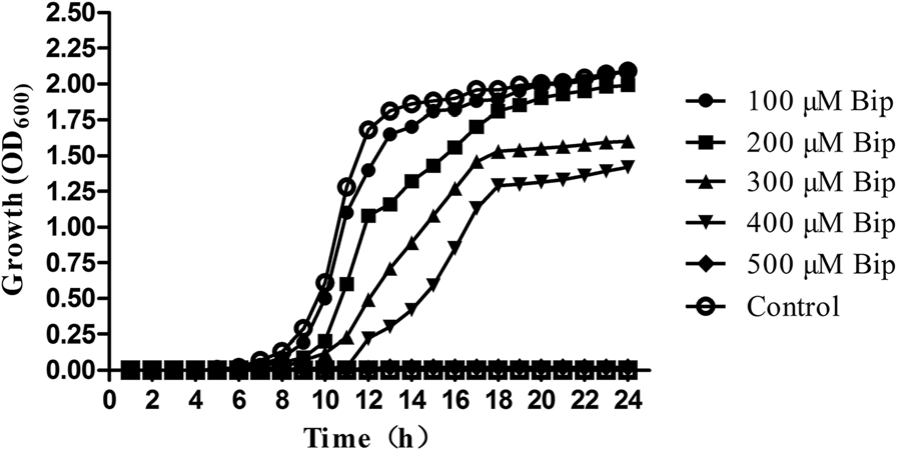
Effect of Bip supplementation on *A. hydrophila* growth. Growth curve (OD_600_) of *A. hydrophila* NJ-35 grown in TSB medium in the presence of 0, 100, 200, 300, 400, and 500 μM Bip

The effects of different concentrations of Bip on the growth of *A. hydrophila* are shown in Fig. 1. When compared with the control group, inhibitory effects were observed in the Bip addition groups, and higher Bip concentrations delayed the time of entering the logarithmic phase and reduced the maximum. When the Bip concentration was 500 μM, the growth of *A. hydrophila* was totally inhibited for at least 24 h. Due to the significant inhibition and higher cells concentration, 200 μM Bip was chosen as the proper iron-limitation concentration for subsequent analyses.

### Expression profile of iron-limited *A. hydrophila*

Based on the transcripts of *A. hydrophila*, 4327 genes were identified and quantified (Table 1). After filtering with FDR, 1204 genes were found to be differentially expressed between the control and iron-limitation groups. Detailed information for most of the DEGs is shown in Table 2. In comparison, the quantity of down-regulated DEGs detected (603) was greater than that of the up-regulated genes (601). A total of 2244 proteins were identified; 2012 were quantified and 1946 were correlated with the transcripts. Additionally, while compared with the control group, a total of 236 DEPs (90 up-regulated and 146 down-regulated) were identified in the iron-limitation groups with an at least 2-fold difference, and 167 of the DEPs were correlated to the corresponding DEGs, which have the same trends. Fewer DEPs are probably due to the removal of some proteins that were secreted by *A. hydrophila* NJ-35 in the supernatant of the experimental design.

**Table 1:** Overall features of the iron-limitation responsive expression profile

Group name	Type	Number of genes	Number of proteins	Number of correlations
Control-VS-Iron-Limitation	Identification	4327	2244	1946
Control-VS-Iron-Limitation	Quantitation	4327	2012	1733
Control-VS-Iron-Limitation	Differential Expression	1204	236	167

**Table 2:** List of differentially expressed genes under iron restriction

Accession	Description	Log_2_FC
U876_09860	Biosynthesis of siderophore group nonribosomal peptides	9.3945
U876_18585	ABC transporters	7.3209
U876_18590	ABC transporters	7.2995
U876_11875	Propanoate metabolism	3.6179
U876_18275	Two-component system|Bacterial chemotaxis	2.7194
U876_05565	Carbon metabolism|Glycolysis / Gluconeogenesis|Citrate cycle (TCA cycle)|Pyruvate metabolism|Butanoate metabolism|Carbon fixation pathways in prokaryotes	2.3607
U876_16675	Quorum sensing	2.3034
U876_14615	Oxidative phosphorylation|Two-component system	2.1593
U876_13185	Ribosome	1.7769
U876_13000	Cysteine and methionine metabolism|Selenocompound metabolism	1.5614
U876_17160	RNA transport	1.4601
U876_00445	Glycine, serine and threonine metabolism	−1.5113
U876_10020	Purine metabolism|Drug metabolism - other enzymes	−1.5726
U876_15390	Biotin metabolism	−2.2592
U876_09705	Selenocompound metabolism|Aminoacyl-tRNA biosynthesis	−3.4550
U876_00975	Biosynthesis of amino acids|Arginine biosynthesis	−3.5694
U876_17185	Lysine degradation|Tropane, piperidine and pyridine alkaloid biosynthesis	−3.8646
U876_15985	Fructose and mannose metabolism|Phosphotransferase system (PTS)	−4.5035
U876_12875	Nitrogen metabolism	−5.4990
U876_00965	Arginine biosynthesis	−6.0546

Note: FC, Fold change, the ratio of different expression levels between the iron-limitation group and the normal TSB group

### Integration analyses of transcriptome and proteome

To identify robust pathways that were corroborated by both datasets, we integrated the differentially expressed transcripts and proteins to find the corresponding genes and proteins, and the results are listed in Additional file 1: Excel S1.

**Fig. 2:**
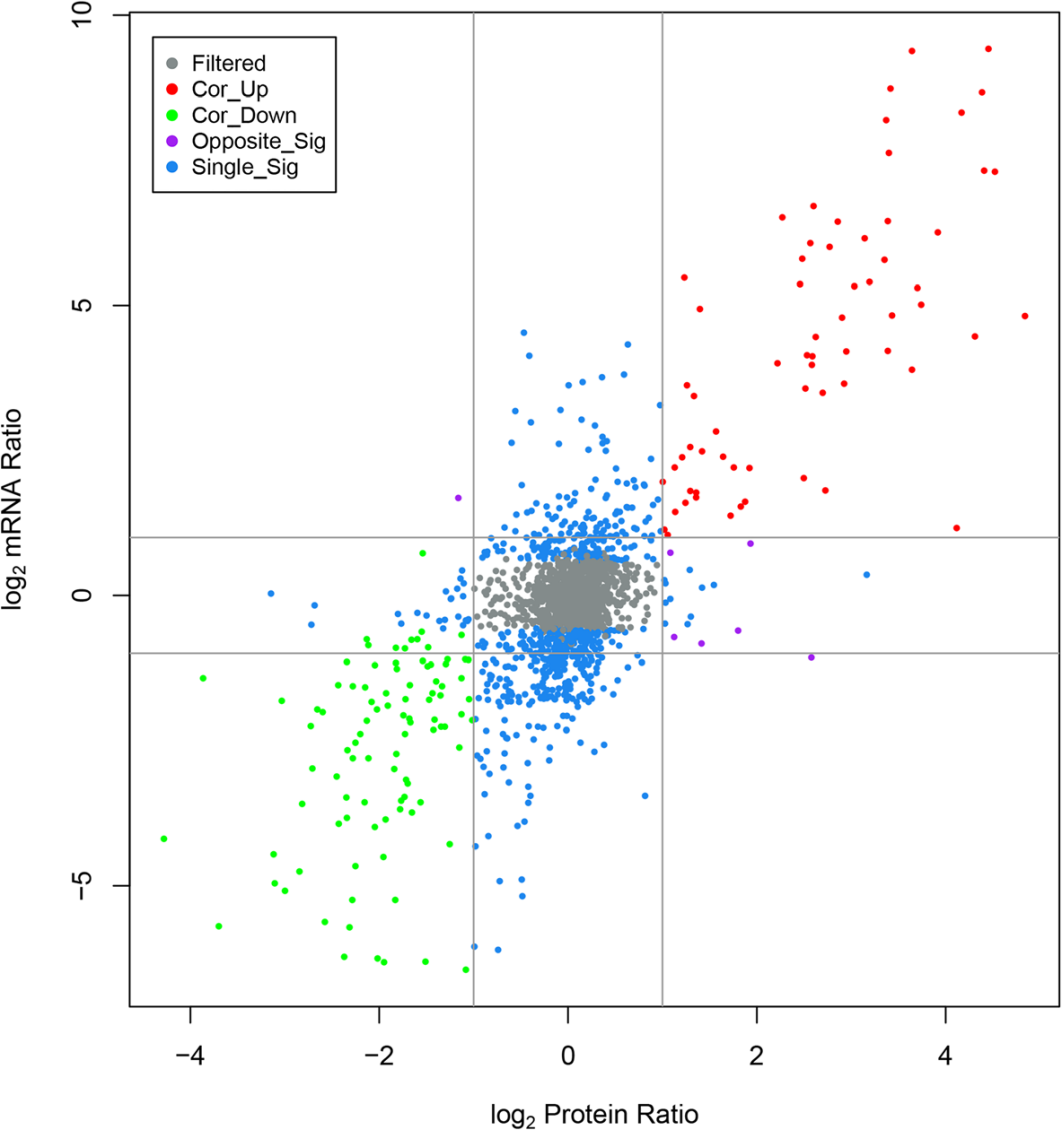
Relationship patterns of all of the quantitative mRNA and protein. In the nine-quadrant diagram, the abscissa is the protein expression and the ordinate is the gene expression. Each color denotes a log_2_ mRNA ratio and a log_2_ protein ratio. Gray (filtered) represents genes and proteins with no significant difference, red (Cor_up) indicates up-regulated genes and proteins, green (Cor_down) indicates down-regulated genes and proteins, purple (Opposite_Sig) indicates that DEGs and DEPs show opposite up- and down- regulation and blue (Single_Sig) indicates that one of the genes and proteins differ

The distribution of the corresponding mRNA: protein ratios is shown in a scatterplot of the log_2_-transformed ratios. As shown in Fig. 2, almost all of the log_2_ mRNA: log_2_ protein ratios are concentrated at the center of the plot, where mRNA and protein levels did not vary above 2-fold. Integration analyses of transcriptome and proteome data revealed that 67 genes and their corresponding proteins were up-regulated, while 94 were down-regulated, reflecting significant changes and showing a strong correlation between the transcripts and proteins. Overall, 680 transcriptomes showed DEGs with no difference in proteins, while 35 transcriptomes showed different proteins but no difference in genes. Conversely, the expression of the following six genes and proteins was opposite (e.g., when the gene was upregulated, the protein was downregulated and vice versa): (U876_04575, YP_857861.1), (U876_17130, YP_855747.1), (U876_17135, YP_855746.1), (U876_19295, YP_855421.1), (U876_20135, YP_855265.1), and (U876_21295, YP_855025.1). This exception can be caused by regulation at several levels, such as post transcriptional processing, degradation of the transcript, translation, post-translational processing and modification. In summary, most of the trends in DEP abundance were consistent with the DEG data.

### Functional classification of enriched DEGs and DEPs by GO and KEGG

**Fig. 3:**
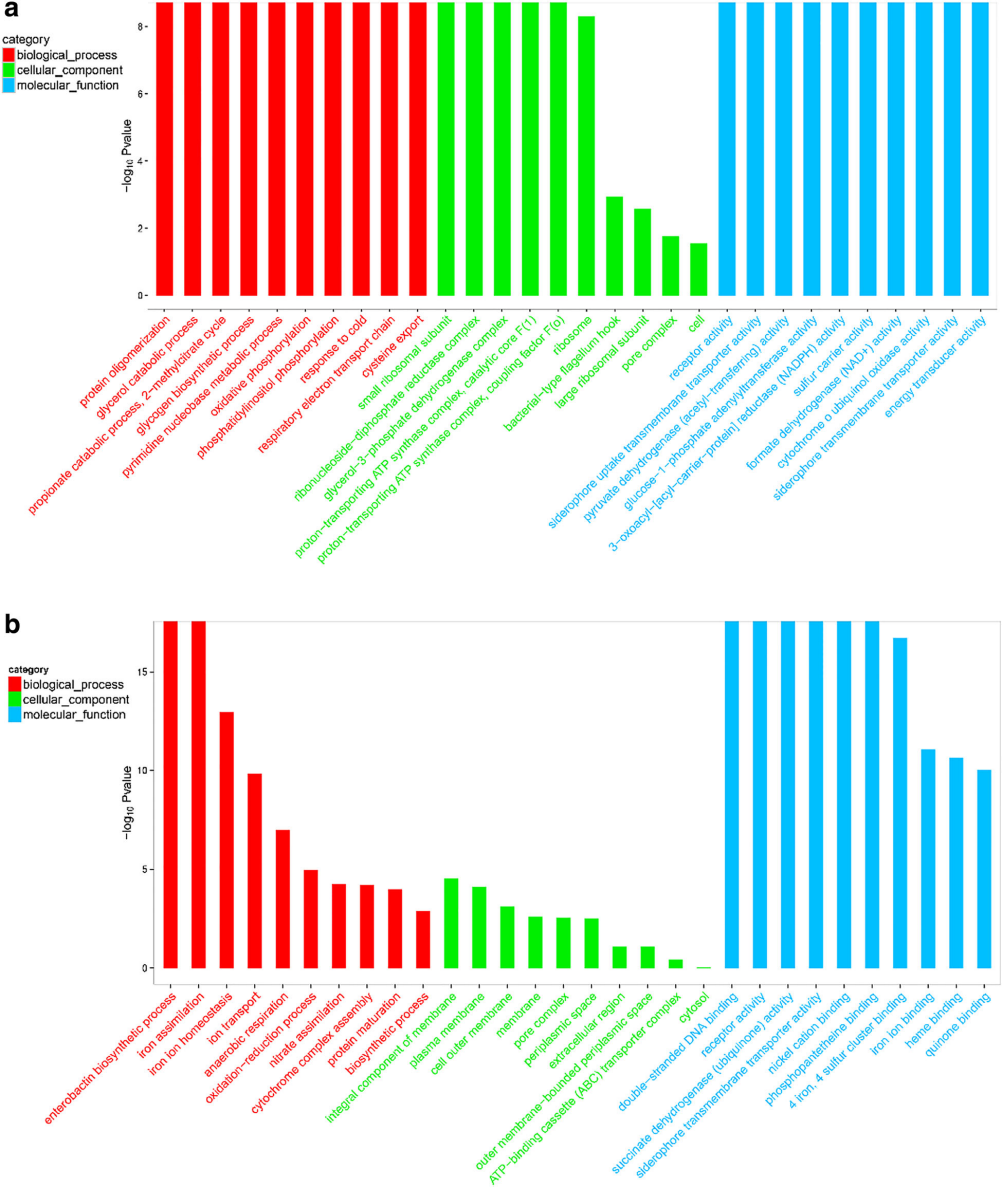
GO enrichment analyses of DEGs and DEPs Control group vs Iron-Limitation group. GO term analyses of transcriptomics (**a**) and proteomics (**b**) that were catalogued as Biological Process, Cellular Component, and Molecular Function

GO enrichment analyses were used to classify the enriched DEGs and DEPs between the control and iron-limitation groups using bioinformatics methods, and the results are listed in Additional file 2: Excel S2 and Additional file 3: Excel S3, respectively. As shown in Fig. 3, the following three ontologies (molecular function, cellular component and biological process) were observed.

DEGs were distributed in up to 1460 GO terms, while DEPs were classified into 402 GO terms. In this case, GO terms related to bacteria energy metabolism, iron ion transport, and virulence. Based on the ‘−log_10_Pvalue’, most of the GO terms in the biological process category were associated with energy metabolism (Fig. 3a and b). Additionally, six genes were categorized as ‘glycerol catabolic process’ (GO: 0019563), three as ‘propionate catabolic process, 2-methylcitrate cycle’ (GO: 0019629), five as ‘oxidative phosphorylation’ (GO: 0006119), and five as ‘respiratory electron transport chain’ (GO: 0022904). Regarding proteomics, DEPs were mainly involved in the synthesis and transport of iron ions and proteins, particularly the following GO terms: ‘iron assimilation’ (GO: 0033212), ‘ion transport’ (GO: 0006811), ‘enterobactin biosynthetic process’ (GO: 0009239), ‘protein secretion’ (GO: 0009306), ‘protein transport’ (GO: 0015031), and ‘electron transport chain’ (GO: 0022900).

In the cellular component category (Fig. 3a and b), three genes were categorized as ‘glycerol-3-phosphate dehydrogenase complex’ (GO: 0009331), five as ‘proton-transporting ATP synthase complex, catalytic core F(1)’ (GO: 0045261), four as ‘proton-transporting ATP synthase complex, coupling factor F(o)’ (GO: 0045263), and seven as ‘bacterial-type flagellum hook’ (GO: 0009424). Regarding proteomics, DEPs were mainly classified in the cell membrane and cytoplasm of GO terms, including ‘integral component of membrane’ (GO: 0016021), ‘plasma membrane’ (GO: 0005886), ‘cell outer membrane’ (GO: 0009279), ‘cytosol’ (GO: 0005829), and ‘cytoplasm’ (GO: 0005737).

In the molecular function category (Fig. 3a and b), 11 genes were categorized as ‘receptor activity’ (GO: 0004872), three as ‘energy transducer activity’ (GO: 0031992), three as ‘cytochrome o ubiquinol oxidase activity’ (GO: 0008827), four as ‘siderophore uptake transmembrane transporter activity’ (GO: 0015344), and three as ‘siderophore transmembrane transporter activity’ (GO: 0015343). Regarding proteomics, DEPs were mainly related to protein activity and binding capacity, including ‘siderophore transmembrane transporter activity’ (GO: 0015343), ‘receptor activity’ (GO: 0004872), ‘iron ion binding’ (GO: 0005506), ‘heme binding’ (GO: 0020037), ‘metal ion binding’ (GO: 0046872), and ‘porin activity’ (GO: 0015288). In summary, GO term enrichment analyses further explained that metabolism, biosynthesis, transmembrane transport and redox homeostasis should be tightly regulated.

**Fig. 4:**
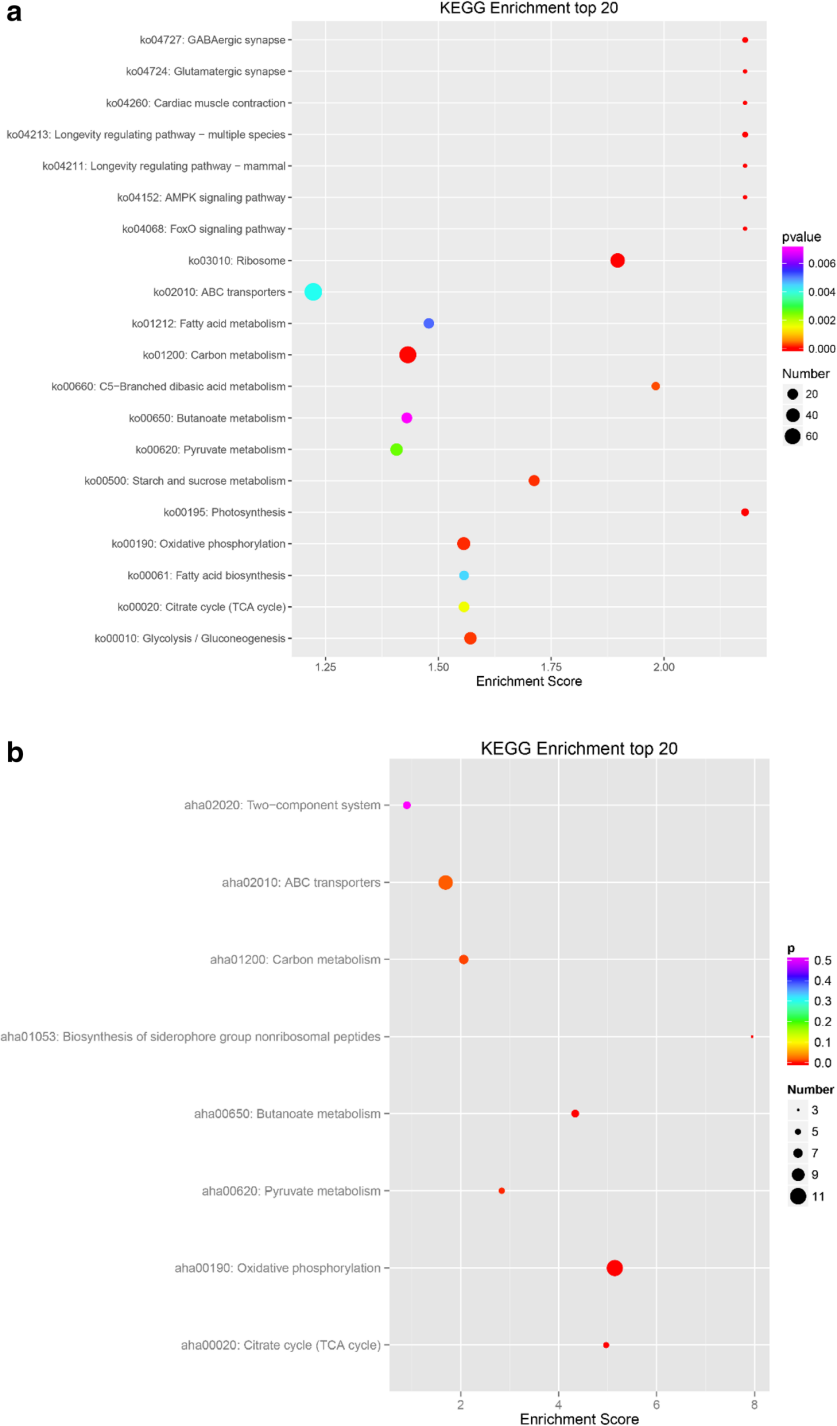
KEGG enrichment analyses of DEGs and DEPs Control group vs Iron-Limitation group. KEGG enrichment analyses of transcriptomics (**a**) and proteomics (**b**)

Enriched KEGG terms are listed under Additional file 4: Excel S4 and Additional file 5: Excel S5, as transcriptomics and proteomics, respectively. When compared with the whole genome, a total of 624 genes were present in the 139 KEGG pathways as DEGs, and we selected the 20 most critical KEGG pathways according to the enrichment scores (Fig. 4a). The up-regulated KEGG pathways included 78 genes under the category of ‘ABC transporters’ (ko02010), 20 genes under ‘TCA cycle’ (ko00020), and 38 genes under ‘quorum sensing’ (ko02024). We inferred that transport, energy production and bacteria interact with each other and may play important roles via stress responses that are regulated through several pathways. The down-regulated KEGG pathways included 47 genes categorized as ‘Ribosome’ (ko03010), 71 as ‘Carbon metabolism’ (ko01200), 31 as ‘Pyruvate metabolism’ (ko00620), and 35 genes as ‘Oxidative phosphorylation’ (ko00190), which confirmed that bacteria slowed down material synthesis and life activities. With respect to proteomics, a total of 41 proteins were detected in the 34 KEGG pathways by DEP, while only eight pathways were found to be significantly enriched by filtration (Fig. 4b). The up-regulated KEGG pathways included three that were labeled under ‘biosynthesis of siderophore group nonribosomal peptides’ (aha01053) and 10 that were labeled under ‘ABC transporters’ (aha02010), indicating clear changes in synthesis and transportation of siderophores. The down-regulated KEGG pathways included 11 proteins that were classified as ‘oxidative phosphorytation’ (aha00190), six as ‘butanoate metabolism’ (aha00650), five proteins as ‘TCA cycle’ (aha00020), five as ‘pyruvate metabolism’ (aha00620), seven as ‘carbon metabolism’ (aha01200), and six as ‘two-component system’ (aha02020), indicating the bacteria repress energy metabolize to adaptive constraint environment. Conversely, the total number of DEPs among them was far smaller than that of the DEGs, and most DEGs and DEPs were down-regulated.

### Clustering of virulence genes and proteins in *A. hydrophila* in iron-limited medium

**Fig. 5:**
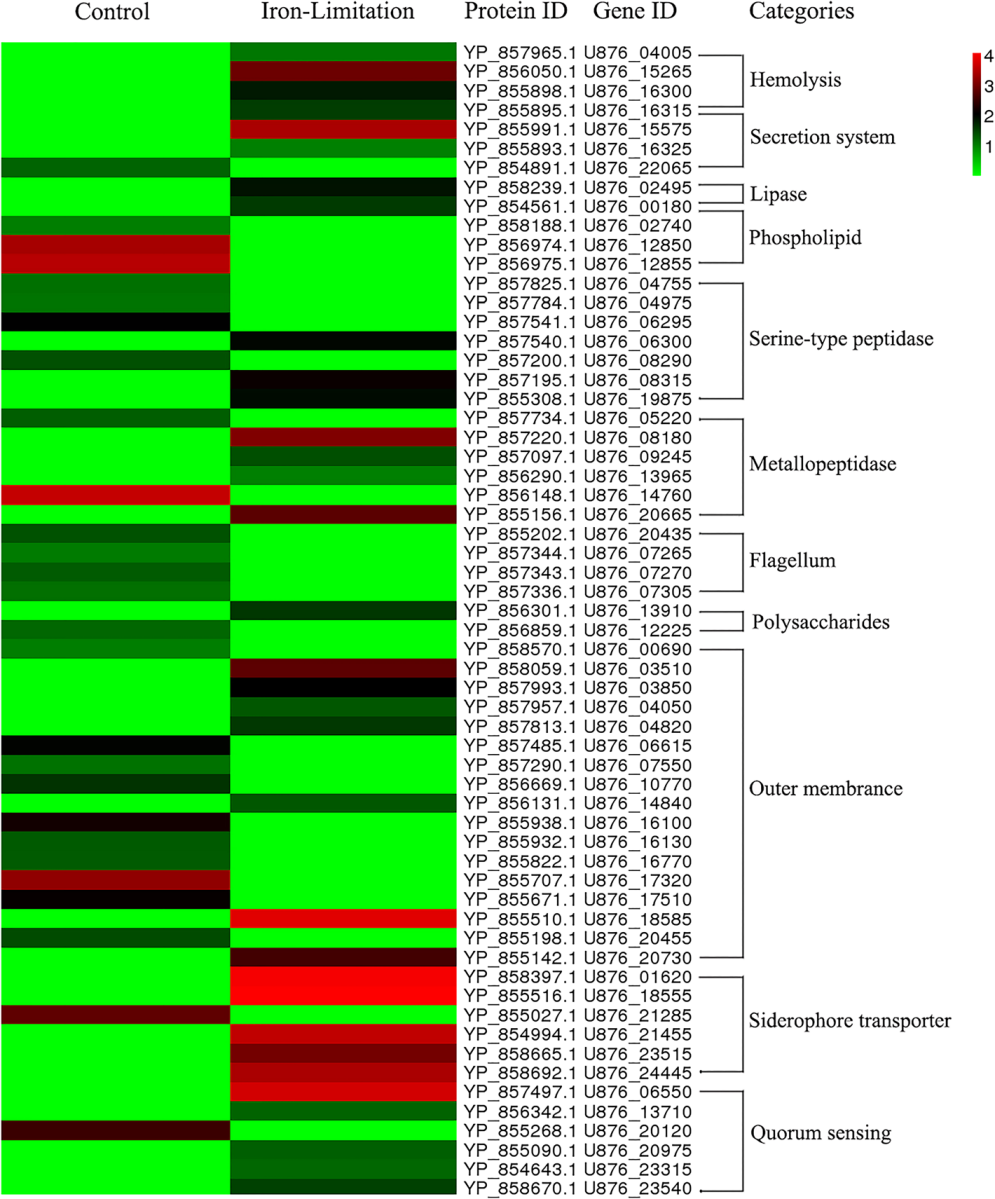
Clustering of 60 mainly related virulence genes and proteins. Numbers are listed as the log_2_ value of difference multiples. Expression differences are shown in different colors; red indicates up-regulation, while green indicates down-regulation. A heatmap was used to visualize the genes and proteins that were related to virulence factor (hemolysis, secretion system, lipase, phospholipid, serine-type peptidase, metallopeptidase, flagellum, polysaccharides, siderophore transporter, quorum sensing, and outer membrane)

According to the bioinformatics analyses, we found that there were 60 virulence factors in the differential genes, which mainly fell under the category of synthesis of iron carriers (U876_01620, U876_18555, U876_21285, U876_21455, U876_23515, and U876_24445), motility of flagella (U876_20435, U876_07265, U876_07270, and U876_07305), and generation of hemolysin (U876_04005, U876_15265, U876_16300, and U876_16315). Heat map analyses (Fig. 5) were used to visualize genes and proteins, and the results indicated a comprehensive impact and clear changes in the regulation of virulence factors.

### Validation of selected DEGs/DEPs by qRT-PCR analyses

To further evaluate the expression of genes in an iron-limited environment, 20 virulence genes (13 up-regulated and seven down-regulated genes) together with reference genes (*rpoB*) were selected for investigation based on their expressions, which were measured by real-time quantitative PCR (RT-qPCR) (Table 3) according to the results of the GO analyses. These selected genes were involved in virulence factors, hemolysis, secretion systems, lipases, phospholipids, serine-type peptidases, metallopeptidases, flagella, polysaccharides, siderophore transporters, quorum sensing, and outer membrane production.

**Table 3:** Primers and sequences used in this study for q-PCR

Name	Gene product	Primer	Sequence (5′➔3′)	qRT-PCR		Illumina	
				Log_2_^FC^	Regulated	Log_2_^FC^	Regulated
U876_04005	RTX toxin	F	GCCAAGAACCTGACCTAC	0.78	Up	1.06	Up
		R	TAACTACCGTCCGACCAT				
U876_15265	hemolysin	F	TGCTCGTACTTGCTGTTG	3.85	Up	3.80	Up
		R	GACTACCTGCTGCTGGAT				
U876_15575	secretin	F	CGATGCGTACCGATATGT	5.00	Up	5.33	Up
		R	AGACTAACAACCAGGATGAG				
U876_16325	type I secretion system	F	GCTCATCGCCTCAATACC	1.42	Up	1.02	Up
	permease/ATPase	R	TAGCCAGTGTGAGTCAGG				
U876_02495	phosphatidylcholine-sterol	F	TTCGGTGTTCCAGCCATA	2.34	Up	1.87	Up
	acyltransferase	R	CCAAGTATCAGGTCATCAAC				
U876_00180	lysophospholipase L2	F	AGCACATAATCGTCAAACTG	1.23	Up	1.51	Up
		R	GCCATCCTCATCGTCAAC				
U876_12850	FAD/NAD(P)-binding	F	CGATTACCACAAGATTGACC	2.34	Down	−5.25	Down
	oxidoreductase	R	TGATCCAGCAGCACTATG				
U876_06295	HPr family phosphocarrier	F	CGGAGACCACAGTGATCT	−0.86	Down	−2.07	Down
	protein	R	TGTACGAGAAGTCTGTTGTT				
U876_06300	cysteine synthase A	F	CAGAGCAATACCCGTGTT	1.69	Up	1.99	Up
		R	TCAACCGTGTTACCAAGG				
U876_14760	peptidase T	F	CCGAGGATCAAACCCATTC	−1.23	Down	−6.23	Down
		R	CTTGCCGTGGAAGTTGTG				
U876_07265	flagellar hook capping protein	F	CAATGTCGGTTACCTGGAA	−0.86	Down	−1.05	Down
		R	GTCCTTGTCCTTGCCATC				
U876_07270	flagellar hook protein FlgE	F	TCAGCGACCTACAGCAAT	0.25	Up	−1.25	Down
		R	CACCAGACAGCAGAGACT				
U876_12225	murein transglycosylase A	F	CCAGACTGATGCCGTAAC	1.25	Up	−1.16	Down
		R	CAAGATGACTCGTCGCTAC				
U876_03850	PAP2 family protein	F	GATGGTGCCGTTGTTCTC	2.54	Up	2.07	Up
		R	ACAGCAGTGGTAGACAGAG				
U876_17510	outer membrane protein	F	GGTGAGTGGAACGGTTAC	−0.99	Down	−2.14	Down
		R	ATCGGAGTGCCAGTAGATA				
U876_18585	hemin ABC transporter	F	CGATCTGGTGCTGGTTAG	4.71	Up	7.32	Up
	substrate-binding protein	R	CTTGATCCACTTGGCGAT				
U876_21455	TonB-dependent siderophore	F	CGTCTCAGTCACCAGTCT	2.61	Up	6.07	Up
	receptor	R	ATCCAGGTTGTTGTTCTTGT				
U876_20975	transcriptional activator	F	TTGAACAGCACCACCTTG	3.33	Up	1.22	Up
	protein AhyR/AsaR	R	GCTTGAGTACCTCGAACAT				
U876_23540	LuxR family transcriptional	F	GAAGGAGTGCCTGTTCTG	1.14	Up	1.45	Up
	regulator	R	TATGATGCCGCTGGAGAT				
U876_09860	2,3-dihydroxybenzoate-AMP	F	TACAGGATGCCGATGGTTA	6.20	Up	9.23	Up
	ligase	R	ATCCGTGCTGACGATGAA				
U876_01300	DNA-directed RNA	F	GGATCACGGTGCCTACAT				
(rpoB)	polymerase subunit beta	R	TAACGCTCGGAAGAGAAGA				

The results of qPCR showed that the majority of the selected virulence factors (90%, 18/20) were consistent with the transcriptome data. Notably, five virulence-related factors, U876_15265 (hemolysin, log_2_FC = 3.80), U876_15575 (secretin, log_2_FC = 5.00), U876_18585 (hemin ABC transporter substrate-binding protein, log_2_FC = 4.71), U876_20975 (transcriptional activator protein AhyR/AsaR, log_2_FC = 3.33), and U876_09860 (2,3-dihydroxybenzoate-AMP ligase, log_2_FC = 6.20) were shown to be significantly up-regulated (log_2_FC > 3.00) under iron-limited conditions. Moreover, two selected genes, U876_07270 (flagellar hook protein FlgE) and U876_12225 (murein transglycosylase A), showed appositive results to the RNA-seq data, which might have been due to differences in the analyses methods.

### Determination of iron concentration

Atomic absorption spectrophotometry revealed that the medium iron concentration of 0.44 mg/100 g in the normal TSB group was higher than 0.28 mg/100 g in the iron-limitation group, indicating that iron scavenger 2,2- bipyridine has a higher efficiency. After bacterial growth, the medium iron content of the normal TSB group was higher than that of the iron-limitation group. Surprisingly, the concentration of 0.664 mg/100 g in the normal TSB group strain cell was lower than 0.998 mg/100 g in the iron-limitation group strain cell. All of the results are shown in Table 4.

**Table 4:** Determination of iron concentration under two culture conditions

Group	Medium before culture/(mg/100 g)	Medium after culture/(mg/100 g)	Strain cell/(mg/100 g)
Normal TSB group	0.44 ± 0.032^b^	0.27 ± 0.029	0.664 ± 0.019^a^
Iron-Limitation group	0.28 ± 0.021^a^	0.20 ± 0.030	0.998 ± 0.012^b^

Note: Means with different lowercase letters within the same column were significantly different (*P* < 0.05)

### Effect of iron-limitation on virulence factors production in *A. hydrophila*

**Fig. 6:**
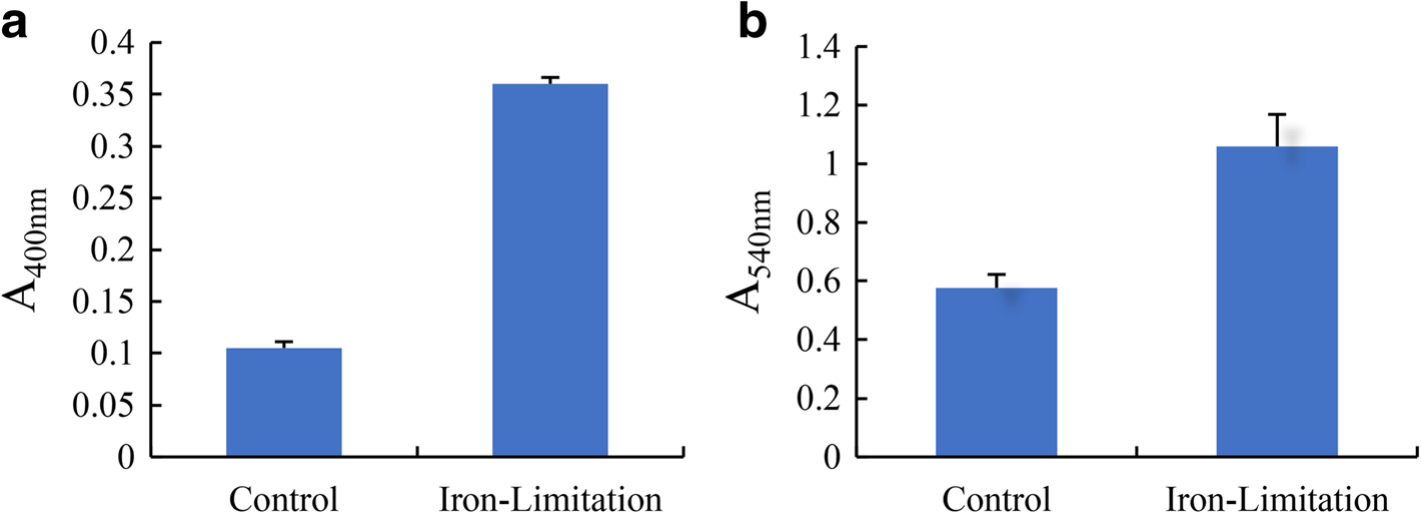
Effect of control and iron-limitation conditions on *A. hydrophila* NJ-35. **a** Total protease, and (**b**) hemolytic activity. The data represent the mean values of three independent experiments and are presented as the means ± SD

As shown in Table 5, the total protease activity in supernatants from *A. hydrophila* NJ-35 growing without Bip was 0.105 (OD400 nm), whereas the presence of Bip resulted in a significant increase in protease activity to 0.36 (OD400 nm) (Fig. 6a). When compared with the control group, the hemolytic activity of *A. hydrophila* NJ-35 was significantly enhanced under iron limitation, indicating that NJ-35 produced 83.8% more hemolysin (Fig. 6b). To observe the hemolysis ability, sheep blood agar plates were used for rough detection. *A. hydrophila* NJ-35 under iron limitation generated a large hemolytic zone on the blood agar plates compared to the control group, but the lipase activity and swarming motility did not differ significantly (Table 5). Interestingly, the swimming ability of the bacteria was strong under iron limiting conditions, which could reflect attempts to move to areas with more suitable conditions (Table 5).

**Table 5:** Effect of iron limitation on *A. hydrophila* extracellular enzyme activity and motility

Virulence	NJ-35	
	Control	Iron-Limitation
Lipase (cm)	0.95 ± 0.16	1.06 ± 0.21
Blood-plate hemolysis (cm)	0.90 ± 0.07	1.07 ± 0.09
Swimming ability (cm)	1.02 ± 0.01^a^	1.17 ± 0.01^b^
Swarming motility (cm)	0.89 ± 0.12^b^	0.84 ± 0.07^a^

Note: Means with different lowercase letters within the same row were significantly different (*P* < 0.05)

### Infection assays

**Fig. 7:**
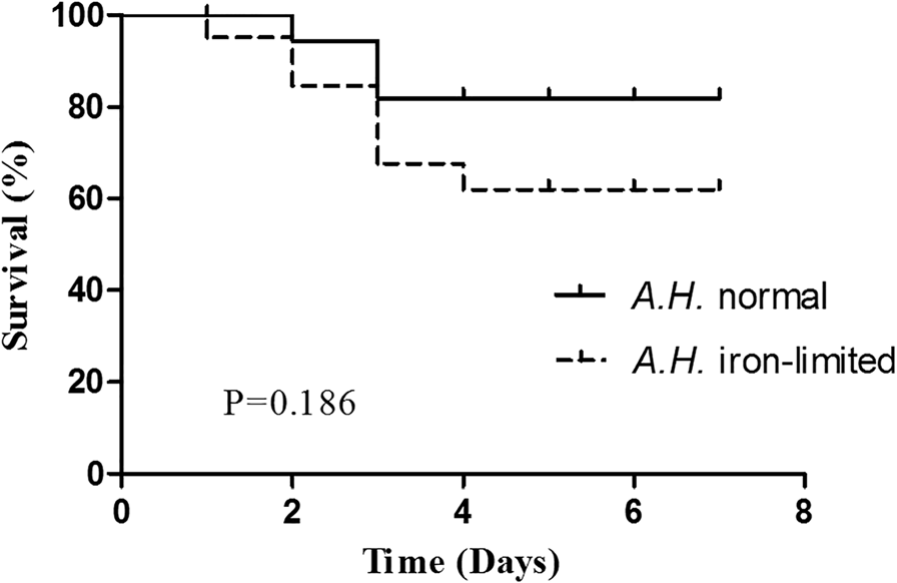
Kaplan-Meier survival analyses of *Megalobrama amblycephala* challenged with *A. hydrophila* NJ-35 from normal and iron-limited media. Data represent accumulative fish mortality in three replicates

The isolated pathogenic bacteria were *A. hydrophila* after morphological, physiological and biochemical, molecular identification. *Megalobrama amblycephala* injected with *A. hydrophila* NJ-35 showed distinct mortality rates under iron and non-iron limited conditions (Fig. 7). Although the difference was not significant, the survival rate in the group injected with *A. hydrophila* was substantially higher (by 19.77%) than that of the iron-limitation group at four days post-challenge.

## Discussion

### Comparative transcriptomic and proteomic analyses

The survival and proliferation of bacteria was sensitive to environment factors. Many environmental stress factors, e.g., pH, temperature, oxygen, acidity and salinity [49, 50] significantly affected the expression of virulence. Iron limitation is an important external stimulus [51] that has profound impacts on almost all bacteria. The culturability and growth rate of *A. hydrophila* were reduced under iron-limited conditions [52]; however, bacterial multiplication was enhanced after injecting exogenous iron into experimentally infected animals, and the virulence of pathogens including *Vibrio cholerae*, *Pseudomonas aeruginosa*, *Klebsiella pneumoniae*, and *Mycobacterium tuberculosis* was established with sufficient iron [8, 53]. *A. hydrophila* establishes virulence through many mechanisms [54], including iron-binding systems, secretion systems, biofilm formation, flagella and pili adhesion, structural proteins, phospholipids, polysaccharides, hemolysis, collagenase, serine protease, metalloprotease, enolase, lipase, and nucleases [5, 6]. The pathogenesis of diseases involves most virulence factors [1], beginning with molecular changes at the micro level and progressing to phenotypic changes at the macro level [55]. Under iron-limited conditions, virulence genes and proteins were up-regulated more than down-regulated (Fig. 5), suggesting that virulence expression was enhanced in *A. hydrophila* to compensate for iron insufficiency, which was confirmed in *F. tularensis* [56]. These virulence factors exerted synergistic effects [57] and contributed to the production of toxins. The results of the infection assays further confirmed this conclusion (Fig. 7). The ferric uptake regulator (Fur) is a negative regulator in iron acquisition systems [58] that controls the expression of 90 virulence and metabolic genes [7, 15, 59]. For example, the biosynthesis of rhizoferring, an iron siderophore in *F. tularensis*, is regulated by operon *fslABCDEF* [60]. In this study, the expression of the *fur* gene (U876_15170) was up-regulated (log_2_FC = 0.3187). This phenomenon could be explained by the higher iron concentration in bacterial cells of the iron-limited group. At the sampling time-point, more iron was stored in the iron-limited group, after which *fur* was up-regulated to reduce the iron absorption [61].

Iron homeostasis was coordinated by the absorption, transport, utilization, and storage of iron ions [62]. *A. hydrophila* utilized multiple iron sequestration systems to hijack host iron ions [63]. Under an iron deficient environment, *A. hydrophila* secreted large amounts of iron transporters and iron-specific scavenger-siderophores. The same results were confirmed by transcriptome analyses of *Bacillus cereus* ATCC 10987, which showed the upregulation of predicted iron transporters in the presence of 2,2-Bipyridine [64]. As an important virulence characteristic of pathogens to both animals and plants [65], siderophores were formed and played a major role in microbial iron acquisition. Siderophore-assisted iron uptake and reductive iron assimilation are both induced upon iron starvation [58]. In previous studies, *A. hydrophila* was found to secrete siderophores to compete with transferrin in vivo to meet the iron demand required for growth and virulence [66]. Measurement of the iron concentration confirmed that the iron chelating ability of bacterial siderophores was notable (Table 4), because *E. coli* [67] and *A. hydrophila* synthesize and secrete enterobactin siderophores [68] in response to iron starvation. Enterobactin synthase subunit E (entE), which is encoded by entA, entB, and entC genes, is a key enzyme involved in the synthesis of isochorismate synthase. In both *E. coli* and *A. hydrophila*, a 22 kB gene cluster including entD-fepA-fes-entD-fepE-fepC-fepG-fepD-fepB-entC-entE-entB-entA-ybdA genes encodes proteins responsible for the synthesis and transport of enterobactin [69]. During this process, the entE polypeptide is responsible for activating the DHBA carboxylate group with ATP by forming the enzyme-bound 2,3-dihydroxybenzoyadenylate as an intermediary in the biosynthetic pathway [70]. Genes with similar enterobactin transport functions (iroN, fepC, cirA, fepC, and iroC) were also found in *Salmonella enterica* [71]. After differential analyses of the genes and proteins, we found that the entE expression level of gene U876_09860 (log_2_FC = 9.39) and protein YP_856992.1 (log_2_FC = 15.46) had increased significantly during the biosynthesis of the siderophore subunits (ko01053). Upon GO term analyses of the DEGs, the entE gene and protein expression levels were not increased significantly, which may have been because of differences in the analyses methods and software. Ferritin is the major iron storage protein in *A. hydrophila* [72]. The data demonstrated that ferritin (U876_00270, log_2_FC = − 0.4088) and bacterioferritin (U876_02285, log_2_FC = 13.4043) participated in iron ion transport and storage, which may benefit the survival of bacteria. The up-regulation of this protein may be responsible for the increased intracellular iron concentration in *A. hydrophila*. The expression levels of bacterioferritin in different isolates, including *F. tularensis*, also varied [73, 74]. The TonB mechanism is essential to the virulence of avian pathogenic *E. coli* [75], indicating that a specific TonB-dependent outer membrane receptor might be involved in the transport of iron from transferrin [76]. TonB-dependent outer membrane receptors TonB-2 (U876_00270, log_2_FC = 5.9844), AHA_4249 (YP_858666.1, log_2_FC = 6.1718), AHA_4250 (YP_858667.1, log_2_FC = 7.4891), and AHA_4251 (YP_858668.1, log_2_FC = 10.7778) were found to be required for the transfer of iron chelators and heme to the periplasm, followed by transport to the cytoplasm by ATP-binding cassette (ABC)-type transporters. Inorganic iron in the periplasm is transported to the cytoplasm by membrane transporters, such as Sfu ABC [77].

Iron influences a number of catalytic reactions involving cell energy metabolism in vivo, including respiration and nucleic acid replication [78]. Overall, when iron demand is not met, some enzymes related to metabolism, the regulation of protein synthesis, and the ability of *A. hydrophila* to utilize nutrients, such as carbohydrates, decreased. It has been hypothesized that decreased virulence might be caused by the loss of metabolic activity and the lack of toxin production [79, 80]. According to bioinformatic analyses conducted in this study, the energy generation system and electron respiration chain appeared to be depressed under iron starvation, which is consistent with previous quantitative proteomic analyses of *A. hydrophila* [24]. Iron scarcity reduces iron utilization in iron nonessential pathways, and limited iron is used for the synthesis of iron-containing enzymes involved in the citric acid cycle and the electron transport chain [81]. For example, the expression of NADP-dependent glyceraldehyde-3-phosphate significantly altered the antioxidant activity of bacteria, and NADPH is involved in the transformation of Fe^3+^ into Fe^2+^ in some of the identified bacteria [16]. Similar to *S. pneumoniae* in manganese limited environments [82], the metabolic activity of bacteria will become inert, so bacteria can survive in these environments for a long time [83]. Based on the high-throughput data analyses, it is apparent that 969 genes decreased, 905 genes increased, 146 proteins decreased, 90 proteins increased, the gene and protein ratio was down-regulated, and the regulation of bacteria itself was also used to interpret iron starvation.

### Virulence evaluation of *A. hydrophila* under iron-limited environment

Many studies have shown that the virulence of *A. hydrophila* increased in response to iron deficiency [52]. Two aspects may contribute to the establishment of bacterial pathogenicity: invasiveness and toxin production [84]. The invasive ability of *A. hydrophila* is closely related to their motility, as well as the secretion of toxins, including aerolysin, hemolysin, and enterotoxin, and extracellular protease. To evaluate the virulence of *A. hydrophila* more comprehensively, we conducted an encompassing study of *A. hydrophila* hemolytic and enzymatic activity in vitro and lethality rate in vivo.

*A. hydrophila* pilus is an important coagulation factor and a major colonization factor that enables bacteria to adhere to host digestive epithelial cells during the invasion process. In terms of virulence establishment, pili-assisted adhesion bacteria were 10–200 times more effective than bacteria that do not express pili [85]. Flagella-mediated motility also promotes the initial stages of adhesion [86]. In this study, although the swimming ability of the control group was significantly stronger than that of the iron restriction group, swimming ability was enhanced under iron-limited conditions, indicating that *A. hydrophila* can overcome unfavorable conditions by accelerating their swimming and adhesion abilities, thereby enhancing their resilience to environmental restraints. Alternatively, these findings demonstrate the complexity of *Aeromonas sp.* virulence.

Lethal pathogenic extracellular products (ECPs) of *A. hydrophila* are produced to compete with rivals for limited iron resources [87]. After removal of ECPs by repeated washing with normal saline, the invasion and pathogenicity of the pathogenic bacteria to the host cells was reduced or even completely lost. As a typical ECP, hemolysin that is synthesized and secreted into the organism’s environment can dissolve various sources of iron by destroying intracellular red blood cells or hydrolyzing hemoglobin. Hemolytic activity was detected both in vivo and in vitro in septic animals, and beta hemolysins isolated from protease deficient strains of *A. hydrophila* were found to lead to the death of catfish [88]. Blood-plate hemolysis results showed that the hemolytic ability of *A. hydrophila* under iron deficiency was stronger than that under normal conditions, and it caused greater toxicity and damage to the host. The results also showed that iron exerted an inhibitory effect on extracellular hemolysin and protease activity. Notability, the presence of hemolysin alone does not cause disease [89].

The invasion of pathogenic bacteria was found to be significantly correlated with the level of corresponding enzyme production, and protease activity [90], which is consistent with the results of this trial. Not only can proteases degrade a variety of proteins to provide amino acids for bacterial survival and growth, but they can also directly cause tissue injury, resulting in the spread through the defense mechanism and evasion of the immune system of the host [91]. In addition, the *A. hydrophila* family of extracellular proteases can cooperate with other virulence factors [92] to activate other pathogenic factors. In this study, *A. hydrophila* NJ-35 under low-iron growth conditions were detected with higher protease activity than the control, demonstrating that iron scarcity can promote NJ-35 virulence factor expression.

## Conclusion

In this paper, we simulated the iron restriction environment in the fish host, coalition analyzed the transcriptome and proteomics data of *A. hydrophila*, and identified the changes of enzyme activity, comprehensively revealed the pathogenicity of *A. hydrophila* increased. This study also provide a profound theoretical basis for the effect of exogenous iron preparation on the toxicity of bacteria.

## Additional files

Additional file 1: **Excel S1.** The results of differentially expressed transcripts and proteins to find the corresponding genes and proteins. (XLSX 87 kb)

Additional file 2: **Excel S2.** The enriched DEGs GO terms between control and iron-limitation groups using bioinformatics methods. (XLSX 42 kb)

Additional file 3: **Excel S3.** The enriched DEPs GO terms between control and iron-limitation groups using bioinformatics methods. (XLSX 42 kb)

Additional file 4: **Excel S4.** The enriched DEGs KEGG terms between control and iron-limitation groups using bioinformatics methods. (XLSX 25 kb)

Additional file 5: **Excel S5.** The enriched DEPs KEGG terms between control and iron-limitation groups using bioinformatics methods. (XLSX 13 kb)

## Abbreviations

ABC: ATPbinding cassette
Bip: 2,2’-Bipyridyl
BLAST: Basic Local Alignment Search Tool
CA: Citric acid
CID: Collision-induced dissociation
DEGs: Differentially expressed genes
DEPs: Differentially expressed proteins
ECP: Extracellular products
FDR: False discovery rate
GO: Gene Ontology
HCD: High-energy collision dissociation
IDA: Information dependent acquisition
iTRAQ: Isobaric tags for relative and absolute quantification
KEGG: Kyoto Encyclopedia of Genes and Genomes
LC MS/MS: Liquid chromatography tandem mass spectrometry
LD_50_: 50% lethal dose
MS: Mass spectrometry
NCBI: National Centre for Biotechnology Information
PBS: Phosphate buffer solution
PRIDE: PRoteomics IDEntifications
QC: Quality control
qPCR: Real-time Quantitative polymerase chain reaction
RIN: RNA integrity value
RNA-seq: RNA Sequencing
SRA: Sequence Read Archive
TCA: Tricarboxylic acid
TSB: Tryptic soy broth
